# Supplementary material for: Nanoscale CAR Organization at the Immune Synapse Correlates with CAR-T Effector Functions
Source: Cells. 2023 Sep 12;12(18):2261. doi: 10.3390/cells12182261 (PMC10527520; doi:10.3390/cells12182261)
Supplement: Supplementary file 1 [file cells-12-02261-s001.zip › cells-2563281-supplementary.pdf]

Supplement figures

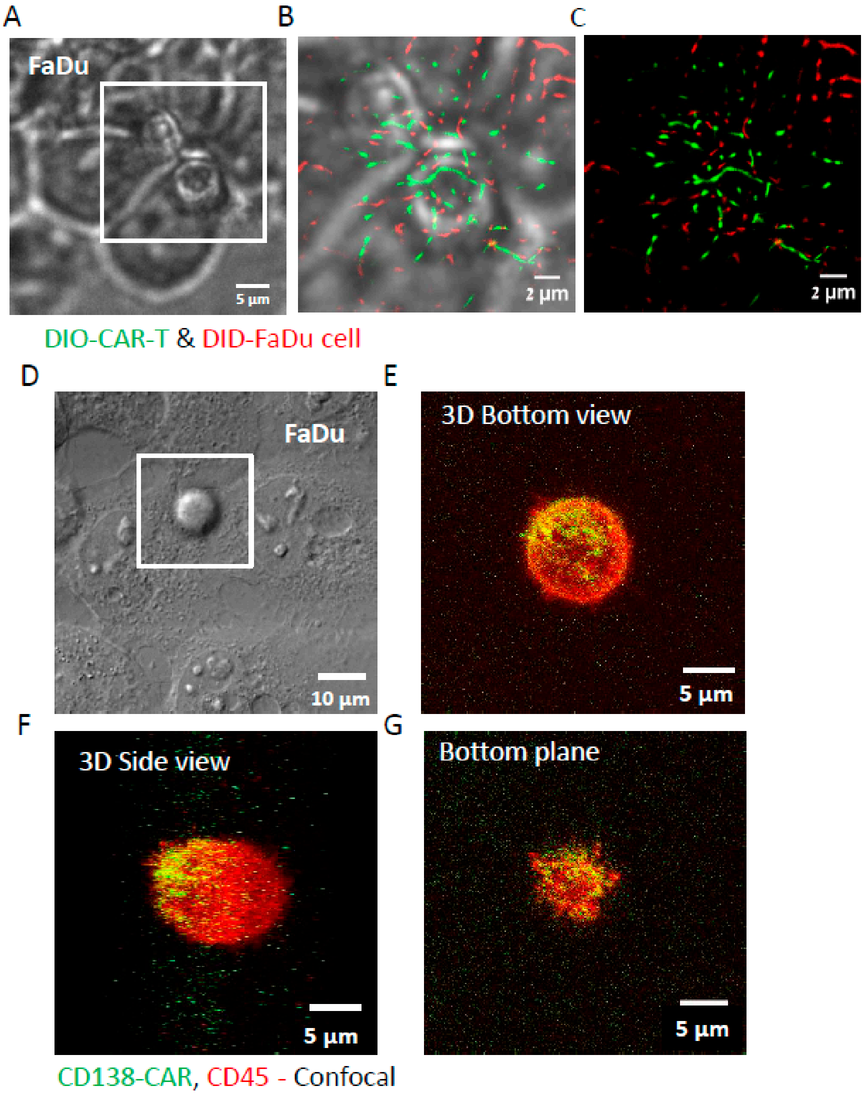

Figure S1. dSTORM and confocal imaging of the interfaces between CAR-T and cancer cells. A-C dSTORM imaging of CAR-T on FaDu cells. The plasma membrane of the CAR-T and FaDu cells was stained with DIO and DID, respectively. **A.** Bright field (BF) image; **B.** A merged, BF and flouresence image; **C.** Zoom in the dSTORM image of panel B on the engagement area. **D–G.** Confocal imaging of CAR-T labled with CAR-Fitc (green) and CD45-Alexa647 (red) Engaing target FaDu cells. **D.** BF image; **E.** Fluorescence image - 3D projection, bottom view; **F.** Fluorescence image 3D projection, side view; **G.** 2D, bottom plane. Scale bars - 5 µm.

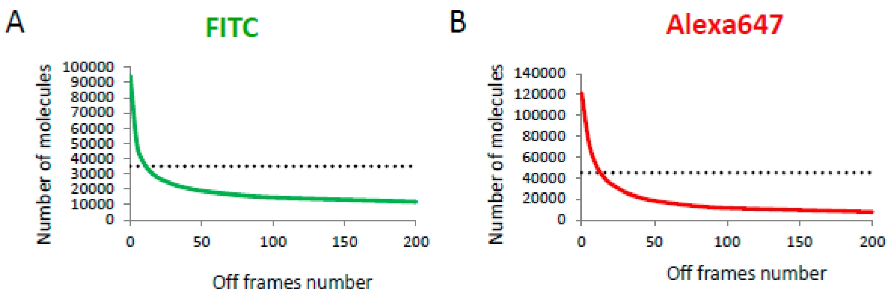

C The values of selected maximum off frames and distance thresholds

	FITC	Alexa647
Maximum off frames	10	45
Maximum distance (nm)	80	100

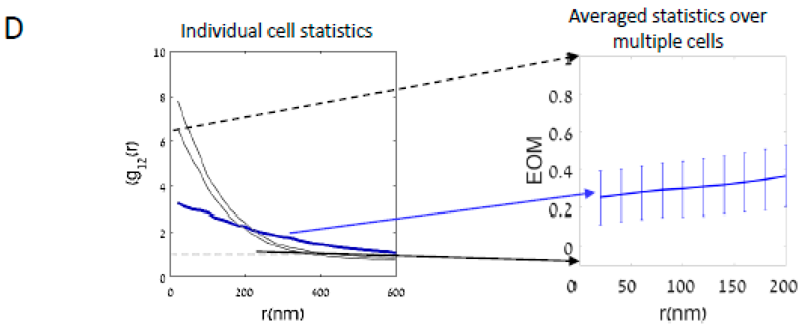

$$EOM^{i}(r) = \frac{g_{12}^{i}(r) - 1}{g_{12}^{RL,i}(r) - 1}$$

Figure S2. Merging parameters for each fluorophore. The number of molecules counted as a function of the temporal gap (number of off-frames) for (**A**) FITC and (**B**) Alexa647. An off-frame threshold (horizontal black line in each plot) was selected for each fluorophore separately according to histograms of the standard-error (sigma) for localizations (see Analyses in Methods). **C.** The values of selected maximum off frames and distance thresholds, used for merging. **D.** Construction of the extent of mixing (EOM) normalizes the bivariate PCF in a way by which a value of 0 corresponds to no interaction, while a value of 1 corresponds to a model of random labelling (a model that indicates high interaction of molecules as they homogenously mix within the studied patterns).

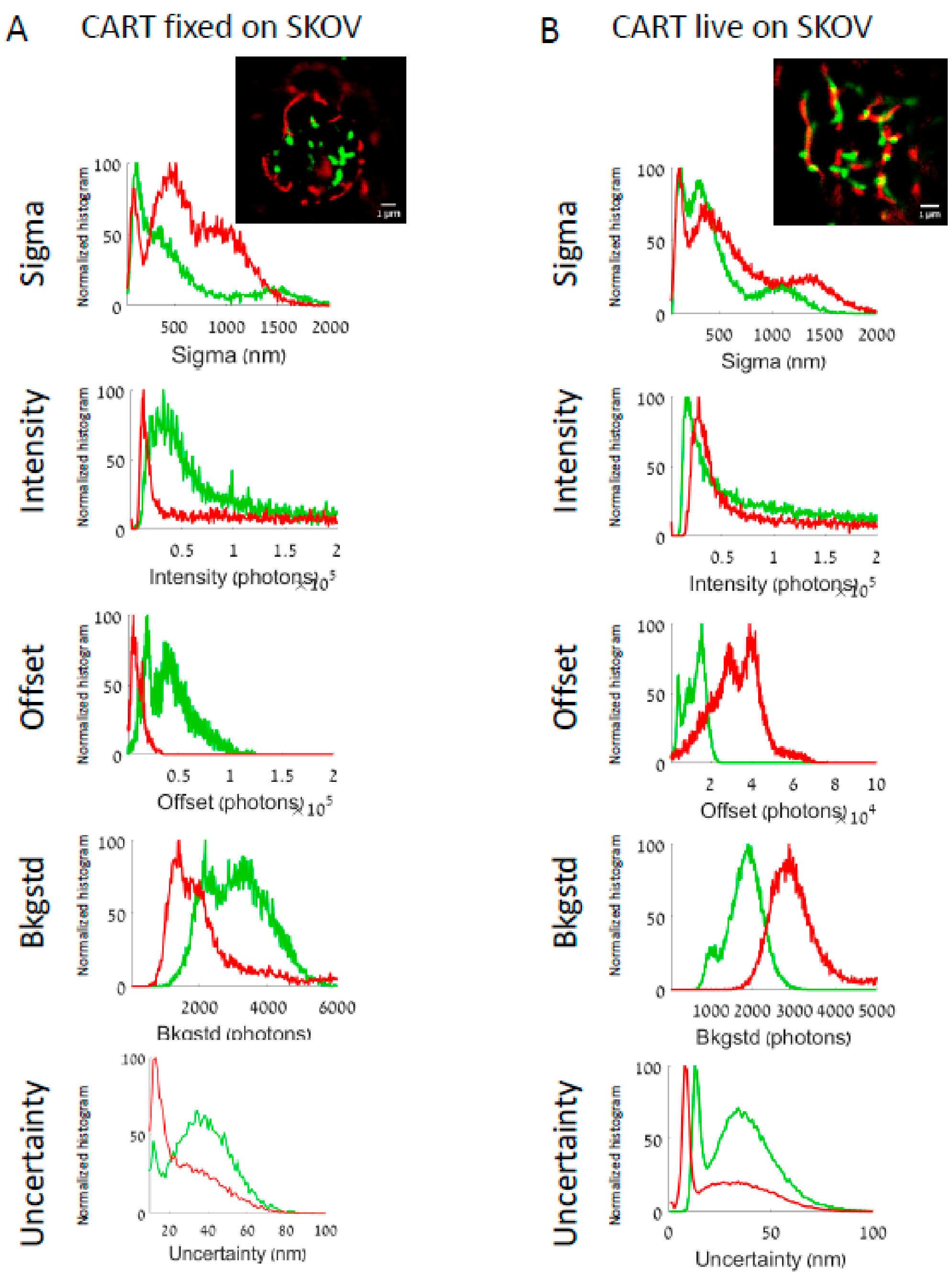

Figure S3. Histograms of imaging parameters. Data of ThunderSTORM analysis of dSTORM imaging of CAR-T cells. **A.** SMLM image of the fixed CAR-T on SKOV-3 cells. CAR-T is labelled with αSTREP-FITC (green) and αCD45-Alexa647 (red). For each fluorophore (green and red), histograms are shown of different parameters. **B.** SMLM image of the live CD138 CAR-T on SKOV-3 cells. CAR-T is labelled with αSTREP-FITC (green) and αCD45-Alexa647 (red). For each fluorophore (green and red), histograms are shown of different parameters. The shown parameters in panels A and B include: Sigma [nm]: standard deviation of the Gaussian fitted to the peak. Intensity [photons]: integrated photons number under the peak (value used to calculate the uncertainty in the Webb/Mortensen formulas). Offset [photons]: baseline of the peak (background absolute value). Bkgstd [photon]: standard deviation of the background (used to calculate the uncertainty in the Webb/Mortensen formulas). Uncertainty [nm]: standard deviation of the lateral localization uncertainty (used to draw the width of each localization when using Gaussian rendering). The number of localizations for each image was: A. Green=11973, Red= 20511, B. Green= 34453, Red= 33435.

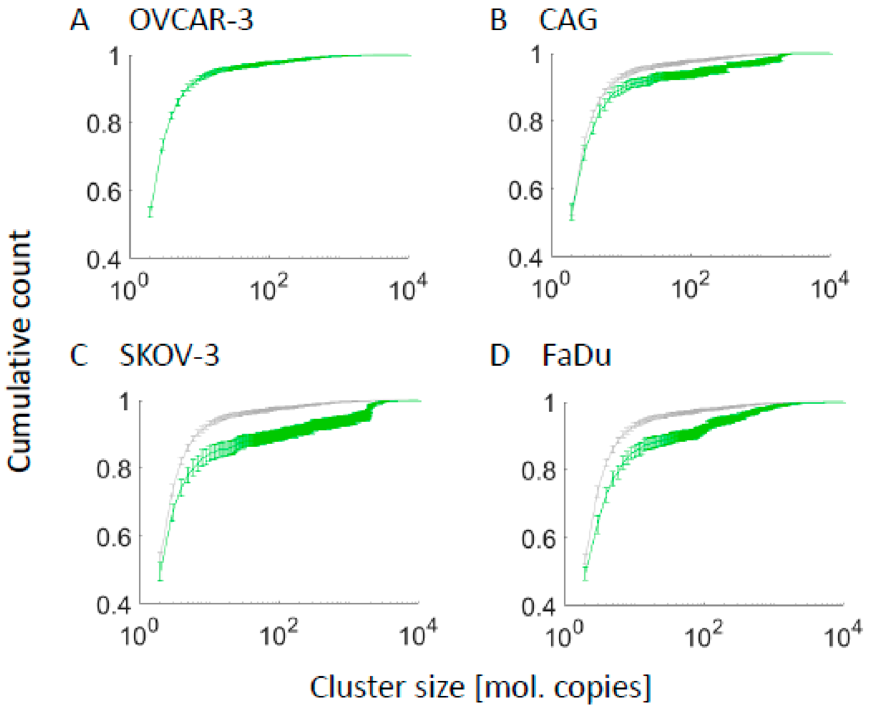

Figure S4. Cluster size analysis of CD138-CAR shows enhanced CAR clustering in target vs. non-target cancer cells. Cumulative histograms of clusters sizes, identified by DBSCAN analysis (see Methods) of CAR in CD138-CAR-T cells engaging OVCAR-3, CAG, SKOV-3 or FaDu fixed cells. **A.** OVCAR-3 (green); **B.** CAG (green); **C.** SKOV-3 (green); **D.** FaDu (green). The results for OVCAR-3 in panels B-D are show in gray for reference.

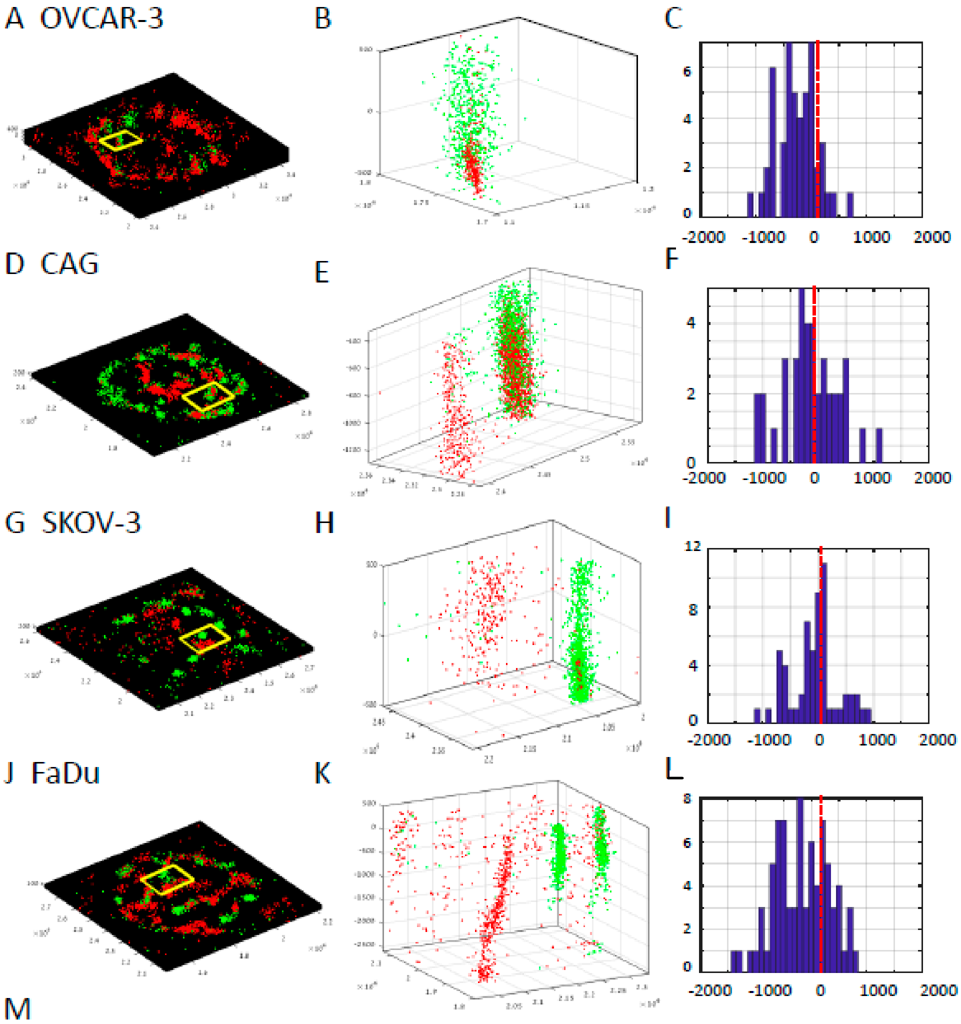

M

	<Δz>	SEM(Δz)	P. value*
OVCAR-3	-262.9	±52	$5*10^{-7}$
CAG	-127	±80	0.1134
SKOV-3	-67	±56	0.2284
FaDu	-167	±56	0.0031

$\Delta z = z(CD45)-z(CAR)$

* Significance of the height difference.

Figure S5. Three-dimensional dSTORM imaging of CAR and CD45 clusters. A–C. CAR-T on OVCAR-3 cells (N=15 cells); **D–F.** CAR-T on CAG cells (N=4); **G–I.** CAR-T on SKOV-3 cells (N=15); **J–L.** CAR-T on FaDu cells (N=15). **A,D,G,J.** Three-dimensional dSTORM images of CAR-T cells on target/non target cells; **B,E,H,K.** Zoom on single clusters of CD45 and CAR from cell images on left (i.e. panels A,D,G,J.); **C,F,I,L.** Histograms of the relative average height of CD45 and CARs in multiple zoom images (Δz = z(CD45)-z(CAR). The value of ΔZ=0 is highlighted by dashed red lines; **M.** Summary of the C45-CAR height difference (namely -   Z, SEM(Z) and the significance of height difference) for each cancer cell line.

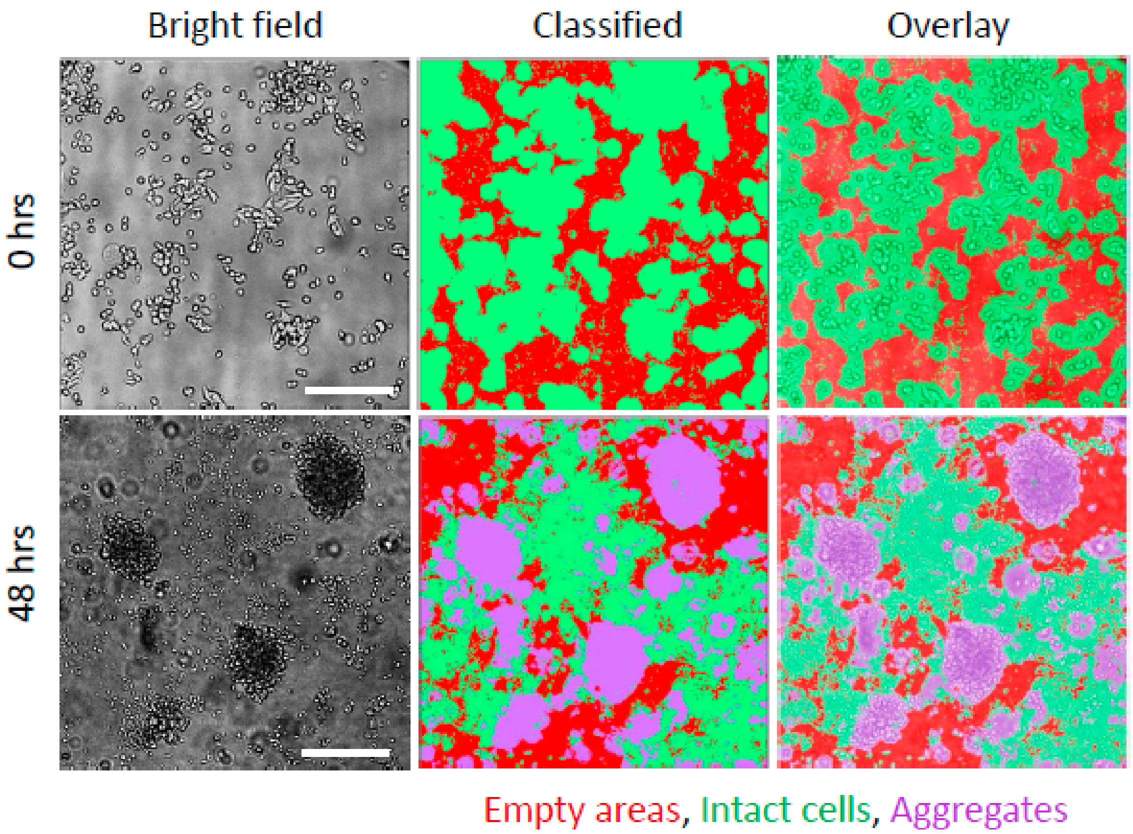

Figure S6. Classification of killed cells in aggregates. Bright-field images of CAG cells before and 24 hrs after engagement with CD138-CAR-T cells. Classification (colored images) was conducted using the Trainable Weka Segmentation (v3.3.1) algorithm in ImageJ, as described in the Methods. Red – empty areas, Green – intact cells and Purple – cell aggregates.
